# Ilmenite Mud Waste as an Additive for Frost Resistance in Sustainable Concrete

**DOI:** 10.3390/ma13132904

**Published:** 2020-06-28

**Authors:** Filip Chyliński, Krzysztof Kuczyński

**Affiliations:** Instytut Techniki Budowlanej, 00-611 Warsaw, Poland; k.kuczynski@itb.pl

**Keywords:** ilmenite mud, waste, concrete, titanium dioxide, frost resistance

## Abstract

Sustainable development leads to the production of building materials that are safer for the environment. One of the ways to achieve sustainability in materials is the addition of industrial wastes and by-products, especially to concrete. However, the addition of waste to concrete often decreases its durability and the scope of aggression of the environment in which the concrete is used has to be reduced. Making sustainable concrete, which is also durable in more aggressive environments, is rather difficult. This article presents the results of tests performed on concrete containing ilmenite mud waste from the production of titanium dioxide, which was exposed to frost aggression with and without de-icing salts. The results have shown that a sustainable and frost resistant concrete can be made. After 200 freeze–thaw cycles, the compressive strength of the tested concretes decreased by less than 4%. Concretes were highly resistant for scaling and after 112 freeze–thaw cycles in water with de-icing salt, the scaled mass was less than 0.02 kg/m^2^. The air void distribution has also been analyzed. The results suited the requirements for frost resistance concrete and were similar to those obtained for a reference concrete with fly ash. The examination of the microstructure using scanning electron microscopy (SEM) has not shown any potential risks that might affect the durability of concrete. Particles of waste were thoroughly combined in the binder and some of its constituents seem to be an active part of the cement matrix. Long-term tests of shrinkage (360 days) have not shown any excessive values that would differ from the reference concrete with fly ash. The presented results have shown that sustainable concrete containing ilmenite mud waste from the production of titanium dioxide might also be resistant to frost aggression.

## 1. Introduction

According to the seventh point of the Basic Requirements for Construction Works CPR-EU 305/2011 published in March 2011, the European Union declares the “sustainable use of natural sources” a priority [1,2]. Following this regulation, while encouraging development, the amount of natural resources used in the production of building materials must decrease as the amount of used by-products and industrial waste must increase. The second aspect of sustainable development is the more effective use of natural sources by producing better materials using the same amounts of constituents only improving their quality; for example, increasing the reactivity of the binder by milling it to smaller particles [3,4]. A third way of making building materials more sustainable is by using recycled building materials from demolitions [5]. Another aspect is that building materials and whole constructions will be more sustainable if the time of usability is extended by more than the typical 50 years, which is the lifetime of most concrete constructions [6].

Adding industrial wastes or by-products might decrease the durability of concrete. In many cases, this is true and new material has to be dedicated to less aggressive environments. In this way, at least some parts of waste are being valorized in order to use less natural sources [7]. If it is possible and safe to use industrial waste as an additive for concrete intended for more aggressive environments, it would be easier to use larger amounts of it. One of the most aggressive occurrences for concrete in moderate climates is frost attack. Concretes projected for such environments have to contain larger amounts of cement, which makes them even less environmentally friendly materials. That is why it is important to also use waste in these types of concretes as well.

The worldwide production of titanium dioxide in 2019 was estimated at 7.2 million tonnes [8]. TiO_2_ is mainly produced through two methods—sulphate and chloride. About 45% of the global production is through the sulphate method, which generates varying amounts of different kinds of by-products and waste. Each tonne of TiO_2_ produced by this method generates about 2.3 tonnes of FeSO_4_·7H_2_O, 1.5 tonnes of FeSO_4_∙H_2_O, 0.7 tonnes of red gypsum, and 0.35 tonnes of ilmenite mud waste [9,10,11]. Iron sulphate is a by-product used mostly as a chromium (VI) reducing agent in the production of cement clinker and as a flocculant in sewage treatment plants. Red gypsum is used in the production of gypsum plasters [10,11,12]. There are only a few publications about the potential ways of valorizing ilmenite mud waste [13,14,15,16], but even when they were successful they could not use large amounts, keeping in mind that the world production of this waste is estimated at 1.1 million tonnes annually [8,17,18].

This article aims to verify the theory that waste material, such as ilmenite mud, might be used as an additive for concrete resistant against freeze–thaw corrosion. This would potentially valorise this industrial waste more widely and make for more sustainable and, thus, greener concrete. Because ilmenite mud waste contains some amounts of unleached TiO_2_ the concrete containing this waste might also have a photocatalytic effect helping to reduce the NOx level in the air [19,20]. The waste probably contains also some amount of nano silica particles which might affect the rheology of cement paste [21,22]. There are two main ways of making a concrete resistant for frost attack. Both of them require a relatively high amount of cement (above 320 kg/m^3^) and low water/cement ratio but one of the way preferred by EN 206 standard [23] requires also entering air into the concrete mix. Air voids prevent the structure of hardened concrete from being damaged by the increasing volume of freezing water [24,25,26,27,28]. The other way of improving concrete’s resistance for frost attack is by making its structure more compacted which prevents the concrete from being penetrated by water and damaged by its freezing. This might be done by using even larger quantities of cement (above 380 kg/m^3^) and low water/cement ratio (0.30 or even less) and without using any air entering agents. This way of protecting concrete from frost attack is more expensive and rather hard as shown in the results of tests performed by Portland Cement Association [29] and others [30] because this type of concrete has a high autogenous shrinkage and might have an early-age shrinkage cracking tendency [31]. This type of frost resistance concrete is being used in the production of prefabricated concrete elements as paving blocks and flags which are made using vibro-pressed technology [32,33,34].

This article presents a new way of valorising ilmenite mud waste as an additive to frost resistance concrete. Previous articles [21,35] have shown that ilmenite mud waste might be a useful material as an additive for typical low cost concretes with low compressive class and made from common materials. This article presents the results of tests performed on higher compressive strength classes, which are durable in more extreme environments including frost attack with de-icing salts.

The article presents the results of the following tests:

- properties of fresh concrete mixes
- compressive and flexural strength
- shrinkage
- frost resistance
- scaling
- air void analysis
- structure examination using scanning electron microscopy (SEM)

As a reference concrete the same concrete mix was prepared but in place of RMUD the same amount of fly ash (FA) class A according to EN 450-1 standard [36], was added. 

Concrete construction depending on its type might be raised with or without reinforcement which affects the properties of used concrete. There are also different types of reinforcements and before using new waste materials in reinforced concrete the suitable tests needs to be performed [37,38]. This article focuses on laboratory tests of concretes without any reinforcement.

## 2. Materials and Methods

Ilmenite mud is a waste created during the production of titanium dioxide by the sulphuric method. The raw feed, containing mostly ilmenite and ilmenite slag, is leached by using concentrated sulphuric acid. Part of the raw material is solubilized and processed further after filtration. Insoluble parts remain, which are called ilmenite mud waste. This waste, classified as hazardous according to European classification [38], is useful as an additive for concrete mostly for its high content of residue sulphuric acid (about 14%). As a result, this waste is additionally rinsed with water and filtered in a factory. After such modifications, the waste contains less than 1% of residual sulphuric acid, which is further neutralized using calcium oxide in the laboratory. Neutralization is carried out until the pH is slightly acidic (about 4–5) to avoid the initiation of pucolanic reaction, as shown in [39]. Next, the neutralization material is dried in the oven at 105 °C until it reaches constant mass. Then, it is sieved through a 0.50 mm sieve. Material prepared in this way is named RMUD (rinsed mud). The results of previous tests have shown that heavy metals present in waste are immobilised in the cement binder at a satisfactory level [40]. Also, the concentration of radioactive nuclides, as some authors have suggested [9,13], is at a safe, low level. 

### 2.1. RMUD, Fly Ash, and Cement

Table 1 and Table 2 present the content of the main constituents received from XRF (X-ray fluorescence) tests and the characteristics of RMUD, fly ash (FA), and Portland cement. The cement used for the tests was CEM I 42.5R Portland cement according to the EN 197-1 standard [41].

### 2.2. Concrete

In order to prepare concrete which will be frost resistant, border parameters were taken from the EN 206 standard [23]. According to this document, concrete that is durable for freeze–thaw cycles in water with de-icing salts has to satisfy the requirements of XF4 and XD3 aggressive environments, where XF is freeze/thaw attack with or without de-icing agents, and XD is corrosion induced by chlorides other than seawater. The border parameters to fulfil these classes of expositions are:

- minimum cement content in the concrete mix: 340 kg/m^3^
- minimum strength class: C 35/45
- maximum water cement ratio (w/c): 0.45
- minimum air entered content: 4.0%
- frost resistant aggregates

**Figure 1:**
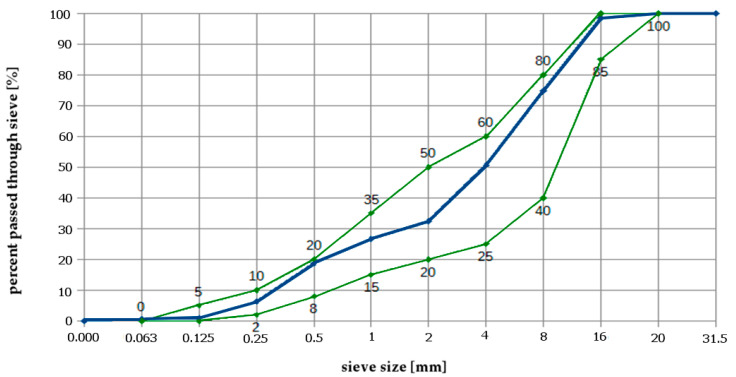
Sieving curves of aggregate mixes used for concretes.

As an aggregate, amphibolite grits fulfilling the requirement of frost resistant aggregates were used. Figure 1 shows the sieving curve of the aggregate mix used in concretes. Border curves (green) are recommended from Polish standard PN-B-06265 [44].

According to previous tests and optimization processes [45], the content of RMUD in concrete should be 10.8% of the binder mass. As a reference concrete, the same mix was used but in place of RMUD the fly ash (FA) has been added. Authors have chosen a reference concrete with fly ash instead of the concrete with only Portland cement as a binder because previous tests have shown [21,40] that the RMUD has a similar level of pozzolanic activity as fly ash.

The composition of concrete mixes is presented in Table 3.

The amount of 340 kg/m^3^ of cement was not enough or the water/binder ratio was too high to fulfil the requirements of the strength class in EN 206 [23] for both of concretes. Increasing the concrete’s compressive strength might be done by increasing the amount of cement or by reducing the water/cement ratio in concrete an adding more of plasticising admixture. In these tests, the compressive strength was increased by adding an additional 10 kg/m^3^ of cement (up to 350 kg/m^3^). 

### 2.3. Properties of Fresh Mix

After mixing the concretes, the properties of the fresh mixes were tested as follows:

- consistency by slump loss method according to EN 12350-2 [46]
- density of fresh mix according to EN 12350-6 [47]
- air content by pressure method according to EN 12350-7 [48]

### 2.4. Compressive and Flexural Strengths

Mixed concretes were placed into 100 mm cubic and prismatic moulds with dimensions 100 × 100 × 500 mm according to EN 12350-1 [49]. The following day, after demoulding, the samples were cured in water at a temperature of 20 ± 2 °C according to EN 12390-2 [50] until the day of the test. Compressive and flexural tests were performed after 28 and 90 days of curing according to the results of previous tests, which have shown that RMUD is a pozzolanic reactive material increasing in composite strength even after 28 days of curing [35,40].

Compressive strength was tested according to EN 12390-3 [51] and a flexural strength test was performed according to EN 12390-5 [52]. The load was put on two points of the samples during the tests.

### 2.5. Shrinkage

In order to check the stability of concrete over time in case there were any expansive reactions in the binder, a shrinkage test was performed using Amsler’s method according to Polish standard PN-B-06714-23 [53] which is similar to the new European standard EN 12390-16 [54]. Three prismatic samples with dimensions 100 × 100 × 500 mm, made of the tested concrete, were measured after demolding up to the 360th day. During the test, the samples were cured at a constant temperature (20 ± 2 °C) and humidity (65 ± 5%) to avoid the influence of the environment on shrinkage.

### 2.6. Frost Resistance

Freeze–thaw tests were performed according to the PN-B-06265 Polish standard [44]. Twelve 100 mm cubic samples were prepared. After curing them for 90 days in water at a temperature of 20 ± 2 °C, six of them were taken for freeze–thaw cycles and the rest were left in the water as reference samples. A total of 200 freeze–thaw cycles were performed. Each cycle included a freezing stage to a temperature of −18 ± 2 °C for at least four hours, and a thawing stage at a temperature of 18 ± 2 °C for two to four hours. After completing the cycles, the samples were examined for any damage on their surface. Next, a test of compressive strength was performed for all 12 concrete samples (including the reference samples) for each type of concrete. According to PN-B-06265 [44], frost resistance concrete in construction with a projected service life of 100 years in variable water levels or contact with de-icing salts has to pass testing after 200 freeze–thaw cycles.

### 2.7. Scaling

**Figure 2:**
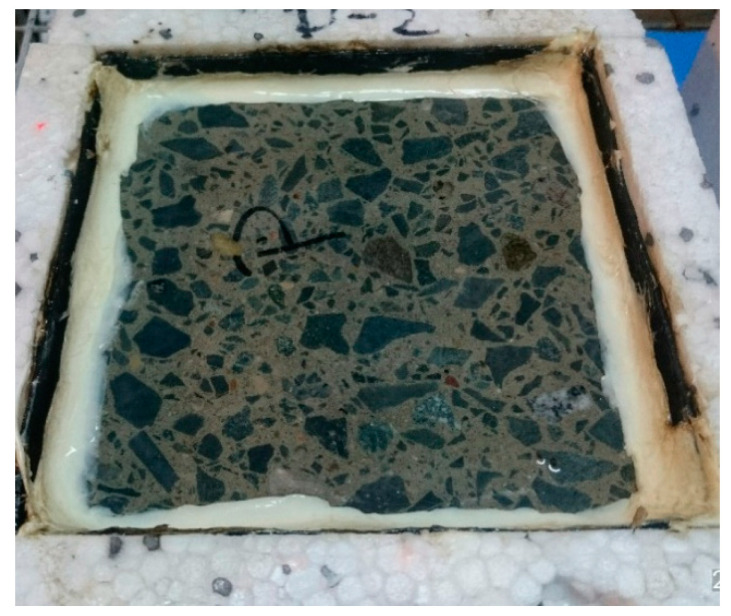
A concrete sample prepared for freeze–thaw cycles.

The freeze–thaw resistance with de-icing salts (scaling) tests was performed according to PKN-CEN/TS 12390-9 [55]. Four 150 mm cubic samples of concrete were cured in water at 20 ± 2 °C for 21 days. After that time, a 50 mm slice was cut from the middle of each, perpendicularly to the surface of mashing. Cut slices were put back to the water until the 90th day of curing. On the 90th day, samples were prepared as shown in Figure 2. On the exposed concrete surface, water with 3% NaCl was poured and a temperature sensor was placed (the level of water was controlled throughout the test). Samples were put into the freezing machine for 112 cycles. Each cycle included a freezing stage to a temperature of −20 °C for two hours and a thawing stage at a temperature of up to 20 °C. One full cycle lasted for 24 h. After 7, 14, 28, 42, 56, and 112 cycles, the samples were taken out and the scaled material was collected from their surface. Then, the samples were put back to the freezing machine with a new portion of the NaCl solution. The collected scaled material was rinsed with water, filtered, dried in the oven and weighed.

### 2.8. Air Void Characteristics

**Figure 3:**
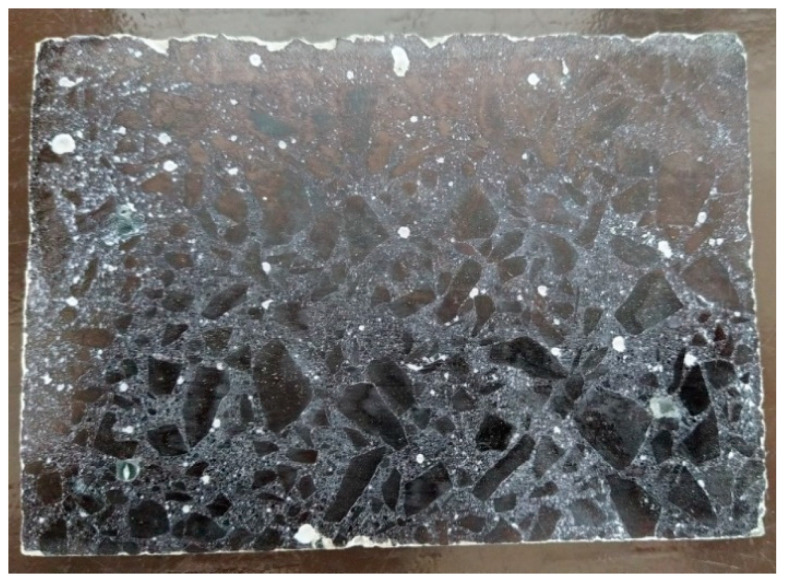
Concrete sample prepared for air pore distribution tests.

The appropriate pore structure in concrete is one of the main aspects of frost resistance concretes [56,57]. Air pore distribution tests were performed according to EN 480-11 [58]. These tests are required by the EN 934-2 standard [59] for air-entraining admixtures. Two 150 mm cubic samples of concrete were cured in water for 14 days after demoulding. Then, from the middle of each, a 10 mm slice was cut perpendicularly to the surface of mashing with a surface size of 100 × 150 mm. The surface of each slice was polished and contrasted after drying. Figure 3 presents how a sample prepared for testing looked like.

Each sample was scanned five times using the Rapid Air 457 automatic air void analysis system.

### 2.9. Scanning Microscopy

Structure observations were made using a scanning electron microscope (SEM) produced by Zeiss, model Sigma 500 VP(Carl Zeiss Microscopy GmbH, Köln, Germany). Secondary electron (SE) and backscattered electron (BSE) images were collected. Phase compositions and mapping were analysed using the EDS detector model Oxford Ultim Max 40 (Oxford Instruments, High Wycombe, UK).

Samples of concrete for microscopic examination were prepared from 90-day-old concrete. First, smaller pieces (20 mm × 20 mm × 5 mm) were cut from the inside of the 100 mm cubic samples. Next, these were dried in the oven at a temperature of 40 °C and put into epoxy resin under vacuum for better filling of the air voids. The final step of preparing the samples was polishing their surface. The samples were gold evaporated before examining them under the microscope. Structure observations were examined only for the RMUD concrete.

## 3. Results and Discussion

### 3.1. Properties of Fresh Mix

Table 4 presents the properties of concrete fresh mixes. The same amount of plasticising admixture was added to both of concretes to reach a proper consistency for moulding the samples (S2–S3 consistency class acc. to EN 206). The air content in both concretes was higher than 4% which suits the border requirements.

### 3.2. Compressive and Flexural Strengths

Table 5 presents the results of the compressive and flexural strength tests of concrete containing RMUD and concrete containing FA. Both samples of concretes reached the projected strength class (C35/45) after 90 days of curing. The strength class was calculated according to EN 206, as per the initial production tests [23].

The results show that the values of both compressive strengths increase between the 28th and 90th day of curing by about 40% for both tested concrete samples. The flexural strength increased to about 6% and 9% for RMUD and FA concrete, respectively. Relatively high increases were observed for compressive strength relating to flexural strength that might be caused by the effect of compacting the microstructure of concretes by the pozzolanic reaction products which increases the compressive strength, but it affects less on the cohesive binding. If cement (CEM I) was the only active constituent in concrete, the compressive strength would remain almost constant after the 28th day [60,61]. This observation proves that RMUD just like fly ash is an active material and plays a role in increasing the strength of concrete. This theory has also been proven in previous tests [35,40].

### 3.3. Shrinkage

**Figure 4:**
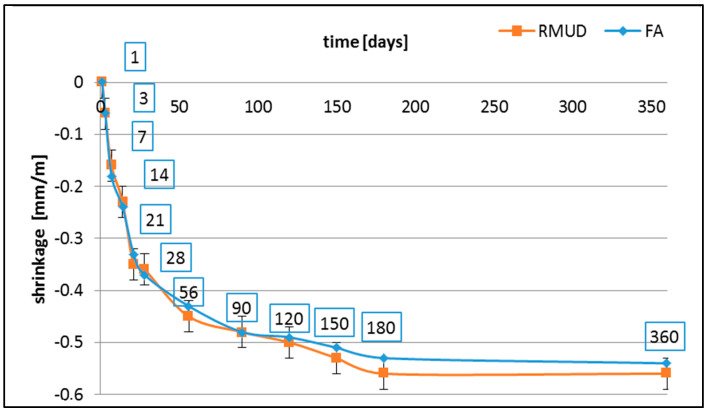
Results of shrinkage tests.

Figure 4 present the results of shrinkage tests. After 120 days, both concretes had almost stopped shrinking, including the uncertainties of the performed test (±0.03 mm/m). No expansion of the samples was observed at any time. The reached value of about 0.5 mm/m and almost the same for both types of concretes is typical for concretes containing these amounts of cement [35,62].

### 3.4. Frost Resistance

After the freezing cycles were completed, concrete samples were weighed and their surfaces were examined for cracks or any other damage. Six of the samples from each of concretes prepared for freeze cycles were weighed before and after the freeze cycles were completed. All 12 samples for each type of concrete (six having undergone freeze cycles and six references) were tested for compressive strength. The results of the freeze–thaw tests are presented in Table 6. 

According to the PN-B-06265 Polish standard, the requirements for frost resistance concrete are as follows [44]:

- no visible damage on the surface of any tested sample
- change of mass in any sample after the freezing cycles cannot be more than 5.0% of the initial mass
- average loss of compressive strength of samples after freezing cannot be higher than 20% compared to the average of the reference samples

The results of frost resistance tests presented in Table 6 have shown that both of the tested concretes suit the above requirements and that they are durable in freeze–thaw environments. After 200 freeze–thaw cycles, there were no cracks on the surface of any specimen nor any other visible damage. Loss of compressive strength of the tested concrete was very low 3.7% and 5.2% for RMUD and FA concrete respectively. The change of mass for both concretes was 0.1%, which is a very good result. This shows that the material should be durable in a frost environment for its projected service life of, at least, 100 years and so is the reference concrete.

### 3.5. Scaling

**Figure 5:**
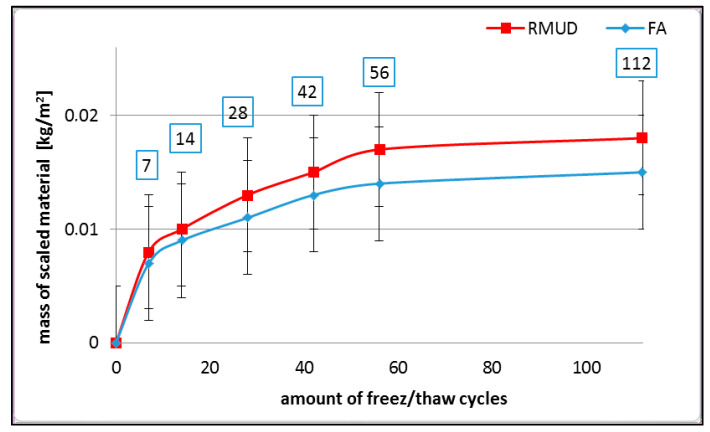
Scaling of tested concrete.

The results of freeze–thaw resistance with de-icing salts (scaling) are presented in Figure 5.

After 112 cycles of freeze–thaw, the mass of scaled material from both types of the tested concretes was less than 0.02 kg/m^2^, which is a very low value compared to the requirements given in EN 1338 [32], according to which the upper layer of concrete paving blocks should not have more than 1.0 kg/m^2^ of the scaled material after 56 freeze–thaw cycles. The recorded values prove that the tested concrete containing RMUD is also durable in a freeze–thaw environment with de-icing agents, such as NaCl, and it is as good as the reference concrete containing fly ash.

### 3.6. Air Void Characteristics

**Figure 6:**
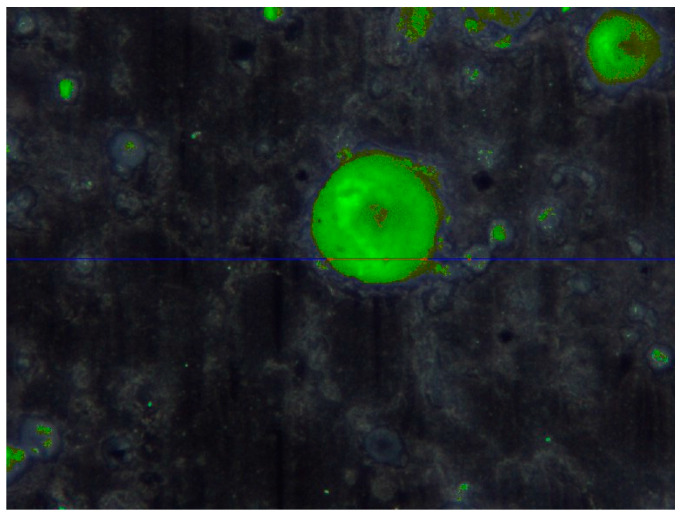
Scanning line of automatic air void analysis system.

Figure 6, presents an example of pictures collected and analyzed by the automatic air void analysis system software. The results are presented in Table 7.

The values of air content obtained in this test are lower than the values received from tests on the fresh mix. This is caused by the fact that an air void analysis does not take into account very large pores (of a few millimeters and above), which does not increase the frost resistance of concrete. The most important air voids, which affect the frost resistance of concrete, are those with a diameter of 300 µm and below. The total air content in those pores (A_300_) above 1% is an appropriate value for frost resistance concretes. The main result from the air void characteristic test is the value of the spacing factor, which is related to the maximum distance of any point in the cement paste from the periphery of an air void. This shows the distribution of air voids in the cement matrix. According to the requirements for air-entraining admixtures given in EN 934-2 [59], the spacing factor should not exceed 200 µm and, according to ASTM C 457 [63], not exceed 230 µm. The values obtained from the tests are presented in Table 7. Both tested types of concretes fulfil both of these requirements. According to the above, both tested concretes should be freeze–thaw resistant.

### 3.7. Scanning Microscopy

In the RMUD concrete sample, leached grains of ilmenite and rutile were spotted. Additionally, particles of almost unreacted plagioclases and pyroxenes, whose surfaces were leached by alkalis from the cement, were observed. Some of the siliceous particles were highly reacted. Siliceous glass phase with trace contents of magnesium, aluminium, sodium, calcium, and titanium was also observed. As relics of clinker, the CA and C_4_AF phases were mostly observed. Magnesium ions which might form the expansive phases were forming the orthopyroxene grains do not affect the durability of the cement matrix. No undesirable reactions which might affect the durability of concrete were spotted.

**Figure 7:**
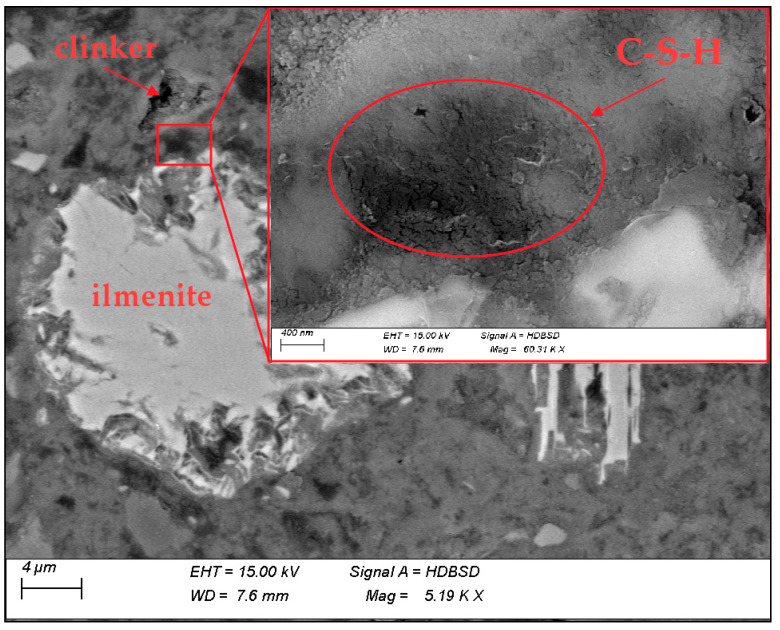
BSE image of concrete.

Figure 7 presents an SEM/BSE image of a leached ilmenite grain and a clinker grain (identified by the EDS analysis). The area between the clinker and the ilmenite grain has been examined for the migration of ions between the ilmenite grain and the CSH phase surrounding the clinker grain.

**Figure 8:**
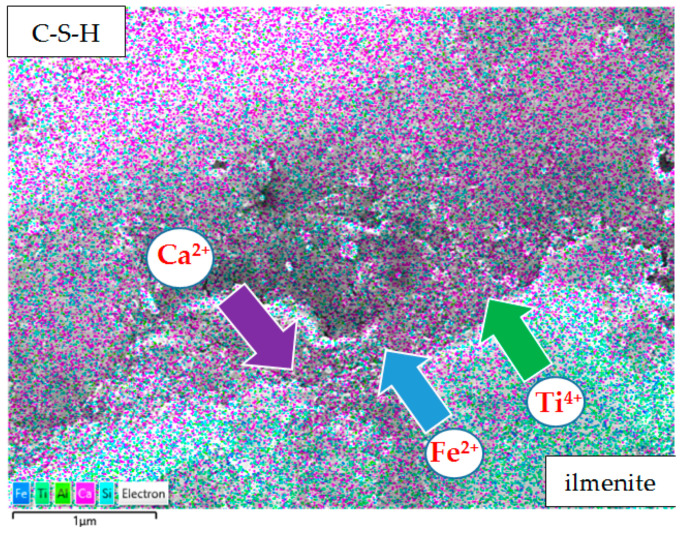
Migration of ions between the C-S-H phase and ilmenite.

Figure 8 presents the area of CSH phase between the clinker and ilmenite grain. EDS mapping shows the diffusion of titanium and iron ions from ilmenite grain into the CSH phase and calcium ions in the opposite direction—from the CSH phase into ilmenite grain. This shows that leached ilmenite grains from RMUD are reactive in the cement matrix and they are an active part of the binder in concrete.

## 4. Conclusions

Upon analysis of the results of the performed tests and comparing them to the results of the reference concrete, the following conclusions have been drawn:

- RMUD waste is an active constituent increasing the compressive strength of concrete between the 28th and 90th day of curing by 40% as was the fly ash in the reference concrete.
- During 360 days of measuring the shrinkage of concrete, no measurements were noted that might suggest that any highly expansive or increasing shrinkage reactions are taking place. The recorded values were almost the same as for the reference FA concrete, which is promising for the durability of concrete.
- Examination of the microstructure of concrete did not show any areas that would suggest reactions that might affect the durability of concrete. Most RMUD particles, as partly leached ilmenite grains and silicon dioxide, were well bounded in the cement matrix. Magnesium ions present in RMUD are constituents of orthopyroxenes and should not affect the durability of cement composites.
- The tested RMUD concrete was highly resistant to freeze–thaw in water and also in water with de-icing salts. The parameters of air void distribution were also satisfactory, which predicts that concrete containing RMUD might be durable in a frost environment for a projected period of 100 years. The results of frost resistance tests were at the same level as for the reference FA concrete. This proves the hypothesis of this article, namely that sustainable concrete containing ilmenite mud waste might also be frost resistant.

## Figures and Tables

**Table 1:** Concentration (%) of main constituents in RMUD, FA, and cement [42].

Element	SiO_2_	TiO_2_	Fe_2_O_3_	MgO	Al_2_O_3_	CaO	Na_2_O	MnO	K_2_O	P_2_O_5_	SO_3_	Cl
RMUD	35.07	33.05	9.65	7.26	5.53	3.09	1.10	0.53	0.26	0.01	0.98	–
FA	51.51	1.09	8.51	2.53	25.71	3.82	1.37	0.10	2.73	0.31	0.48	0.02
Cement	20.06	–	3.38	0.89	4.13	64.41	0.24	–	0.56	–	2.97	0.07

**Table 2:** Physio-mechanical characteristics of cement, RMUD, and FA [39].

Characteristic	Value
**Cement**	
Loss on ignition (%)	4.74
Insoluble residue (%)	0.89
Density (g/cm^3^)	3.05
Relevant surface (cm^2^/g)	4060
Compressive strength (MPa) acc. to EN 196-1 [43]:	−
−2 days	29.2
−28 days	54.2
Bending strength (MPa) acc. to EN 196-1 [43]:	−
−2 days	5.4
−28 days	7.9
**RMUD**	
Loss on ignition (%)	2.70
Relevant surface (cm^2^/g)	8.390
Density (g/cm^3^)	3.15
**FA**	
Loss on ignition (%)	1.43
Relevant surface (cm^2^/g)	4020
Pozzolanic activity (%) acc. to EN 450-1 [36]:	−
−28 days	77.4
−90 days	93.3
Density (g/cm^3^)	2.20

**Table 3:** Composition of tested concretes.

Constituent	Quantity (kg/m^3^)
Portland cement CEM I 42.5R	350
RMUD or FA	42 (10.8% b.m.) ^1^
Aggregate 0/2 (rinsed mining sand)	478
Aggregate 2/8 (crushed amphibolite)	511
Aggregate 8/16 (crushed amphibolite)	730
Water	176 (w/b = 0.45)
Air entraining admixture	1.37 (0.35% b.m.) ^1^
Plasticising admixture	0.67 (0.17% b.m.) ^1^

^1^ b.m.—binder mass (mass of cement + mass of RMUD).

**Table 4:** Properties of concrete mix.

Property	RMUD Concrete	FA Concrete
Slump loss (mm)	110 ± 10(S3) ^1^	80 ± 10(S2) ^1^
(consistency class acc. to EN 206)		
Density of concrete mix (kg/m^3^)	2,340 ± 20	2390 ± 20
Air content (%)	5.4 ± 0.5	4.8 ± 0.5

^1^ class of consistency according to EN 206.

**Table 5:** Compressive strength of concretes.

**Concrete**	**Average Compressive Strength (MPa)**	**Standard Deviation (MPa) (Coefficient of Variation)**	**Compressive Strength Class Acc. to EN 206**
RMUD 28 days	36.2 ± 2.0	2.1 (0.06)	C25/30
RMUD 90 days	51.2 ± 2.0	1.7 (0.03)	C35/45
FA 28 days	35.7 ± 2.0	2.3 (0.06)	C25/30
FA 90 days	49.5 ± 2.0	0.7 (0.01)	C35/45
−	**Average Flexural Strength (MPa)**	−	−
RMUD 28 days	6.6 ± 0.3	0.2 (0.03)	−
RMUD 90 days	7.0 ± 0.3	0.1 (0.02)	−
FA 28 days	6.3 ± 0.3	0.3 (0.04)	−
FA 90 days	6.9 ± 0.3	0.4 (0.06)	−

**Table 6:** Results of freeze–thaw tests (200 cycles).

Samples	Average Compressive Strength (MPa)	Standard Deviation (Coefficient of Variation)	Average Compressive Strength (MPa)	Standard Deviation (Coefficient of Variation)
–	RMUD concrete		FA concrete	
**Reference samples**	56.8	0.96 (0.02)	59.6	1.59 (0.03)
**Samples after freeze–thaw cycles**	54.7	1.19 (0.02)	56.5	1.91 (0.03)
−	**Loss of compressive strength after 200 cycles of freeze–thaw (%)**			
−	3.7		5.2	
−	**Loss of mass after 200 cycles of freeze–thaw (%)**			
−	0.1	0.03 (0.37)	0.1	0.04 (0.38)

**Table 7:** Results of the air pore distribution test.

Characteristics	Average	Standard Deviation (Coefficient of Variation)	Average	Standard Deviation (Coefficient of Variation)
−	**RMUD Concrete**		**FA Concrete**	
**Spacing factor l (µm)**	152.9	9.1 (0.1)	151.7	15.0 (0.1)
**Air content (%)**	2.77	0.33 (0.12)	3.51	0.65 (0.18)
**Micro air content A_300_ (%)**	1.19	0.14 (0.11)	1.64	0.42 (0.26)
